# Voluntary Blood Donation in Modern Healthcare: Trends, Challenges, and Opportunities

**DOI:** 10.3390/epidemiologia5040052

**Published:** 2024-12-17

**Authors:** Svjetlana Gasparovic Babic, Antea Krsek, Lara Baticic

**Affiliations:** 1 Teaching Institute of Public Health of Primorsko-Goranska County, 51000 Rijeka, Croatia; svjetlana.gasparovic-babic@zzjzpgz.hr; 2 Faculty of Health Studies, University of Rijeka, 51000 Rijeka, Croatia; 3 Faculty of Medicine, University of Rijeka, 51000 Rijeka, Croatia; tea.krsek@gmail.com; 4 Department of Medical Chemistry, Biochemistry and Clinical Chemistry, Faculty of Medicine, University of Rijeka, 51000 Rijeka, Croatia

**Keywords:** blood donation, health promotion, public health

## Abstract

Voluntary blood donation plays a crucial role in public health by ensuring a stable supply of blood and its components, essential for medical treatments including surgeries, trauma care, and chronic disease management. Voluntary donors, often healthier individuals, enhance the safety of the blood supply and play a vital role in emergency preparedness. Beyond its societal benefits, regular blood donation may offer health advantages for donors, including improved cardiovascular health due to reduced iron levels and psychological benefits stemming from altruism and a sense of social responsibility. Public health initiatives are instrumental in fostering blood donation through education, awareness campaigns, and targeted donor recruitment strategies. These efforts encourage a culture of social responsibility, inspiring community participation and improving overall healthcare system sustainability. Despite these efforts, challenges such as fluctuating donation rates, aging populations, and shifting demographics underscore the need for ongoing initiatives to ensure a sufficient blood supply. As a public health priority, voluntary blood donation demands continuous promotion and innovation to meet the growing demand for blood products and maintain healthcare system resilience. This review highlights the public health significance of voluntary blood donation, with particular focus on its benefits for donors and its role in supporting healthcare systems.

## 1. Introduction

Blood donation is the voluntary process of providing a portion of one’s blood for medical purposes, either for direct transfusion or the production of blood products. This life-saving act plays a critical role in healthcare, providing essential support for patients undergoing surgical procedures, managing chronic illnesses, or recovering from traumatic injuries [1]. Blood donation can be performed in several ways: whole blood donation, where the entire volume of blood is collected; platelet donation, which focuses specifically on collecting platelets; and plasma donation, which involves collecting the liquid component of blood. Unlike paid blood donation systems, voluntary donation is rooted in altruism, emphasizing community support and a shared responsibility for safeguarding public health [2].

Voluntary blood donation forms an integral part of the overall healthcare system worldwide in making a steady and safe blood supply available according to needs related to treatments of various conditions, including trauma, cancer, and chronic diseases. The World Health Organization (WHO) asserts that voluntary blood donation is not only a means of maintaining health; it is an act of commitment concerning community health and social bonding [3]. In the last couple of decades, efforts have been intensifying to improve the voluntary donation culture due to a fast-growing demand for blood and its related products among seasonal fluctuation and emergency response needs [2].

Therefore, blood donation has some implications for public health. While it helps in the immediate care of casualties in emergencies such as natural disasters and other multiple casualty incidents, voluntary donation also aids the patients who have to be kept under constant medical attention. This includes, in particular, people with rare blood types or suffering from chronic illnesses that necessitate periodic blood transfusions [1]. Moreover, a regular blood supply provides an opportunity for health systems to respond promptly in case of emergencies; therefore, there is a need for continued donor participation. Research has documented that voluntary blood donors are usually healthier individuals, adding to the safety profile of the blood supply. Studies highlight that an all-inclusive education program for donors should be provided to eliminate myths and make them more aware of the need for blood donation. These activities introduce an atmosphere of selflessness and social responsibility, whereby persons are prompted to be concerned with the lives of others and that of society in general [1,2].

A voluntary, unpaid blood donor is any healthy person who donates blood, plasma, or other blood components voluntarily and without any form of payment, either monetary or otherwise. Blood donation is a privilege for healthy people because only a healthy person can be a blood donor [4]. In this context, it is clear that blood donation is a public health priority. Since educating the population, health education, and promoting health culture are within the public health domain, they can also be implemented in promoting blood donation.

For example, public health campaigns can highlight the importance of blood donation through various media channels [5,6]. Community health fairs can include blood donation drives where people can learn about the donation process and its impact. Schools and universities can organize informational sessions and donor drives to engage young and healthy individuals. In education, it is important to accurately inform potential voluntary blood donors about what blood donation is, the risks involved, and the benefits of blood donation. Even individuals who cannot donate blood due to permanent disqualification because of their health status can contribute by promoting voluntary blood donation within their communities [3,4,5,6].

The aim of this review was to provide a comprehensive summary of all aspects of blood donation. This includes an in-depth exploration of the benefits, such as saving lives, supporting medical treatments, and fostering a sense of community and altruism among donors. Additionally, the review delves into the potential risks associated with blood donation and other donor-specific concerns. It also examines different characteristics of blood donation programs worldwide, highlighting their operational frameworks, eligibility criteria, and strategies to ensure donor safety and an adequate blood supply for medical needs.

## 2. Blood Donation: Challenges and Opportunities

Institutes and clinics specializing in transfusion medicine, along with all entities involved in organizing blood donations, must adhere to stringent professional standards to ensure the safety and well-being of both donors and recipients [5]. Recognizing the pivotal role of transfusion medicine, the World Health Organization (WHO) recommends that all activities related to blood collection, testing, processing, storage, and distribution be managed at the national level through a well-coordinated and integrated blood supply network. A robust national blood system should be guided by a comprehensive blood policy and supported by a legal framework to guarantee the consistent application of safety and quality standards for blood and blood products.

The WHO offers policy guidance and technical support to countries to ensure that everyone has access to controlled blood and blood products. It also promotes the goal of achieving self-sufficiency in safe blood supply through voluntary, unpaid blood donations, aligning with its broader objective of “Health for All” through universal health coverage [6].

In the European Union (EU), blood donation and transfusion services are coordinated at both national and regional levels. The chain of blood supply may follow a national, hospital-based, or hybrid system [7]. To ensure compliance with EU regulations, the entire process of blood donation, as well as the blood and blood products themselves, are strictly monitored and tested for infectious diseases such as HIV, hepatitis B, hepatitis C, syphilis, and the West Nile virus. This testing is crucial for preventing the transmission of infections through blood transfusions.

Despite the recognized importance of blood donation, the EU continues to face challenges in maintaining an adequate blood supply. Issues such as an aging population, changes in lifestyle, and the impact of the COVID-19 pandemic have influenced donor availability, making this area of healthcare a complex and ongoing concern [8]. During the COVID-19 pandemic, the storage of frozen blood components proved to be a critical strategy for maintaining blood supplies during periods of fluctuating donation rates and increased demand for certain blood products. Frozen components, such as cryopreserved red blood cells and plasma, served as a vital reserve when traditional blood donation channels were disrupted due to lockdowns, donor hesitancy, and staff shortages. This approach underscored the importance of enhancing blood storage technologies to improve the longevity and availability of blood products, particularly during global emergencies.

The pandemic significantly affected voluntary blood donation, with many countries reporting an initial decline in donor turnout due to movement restrictions and fear of infection. Public health campaigns were rapidly deployed to reassure donors about safety measures, including enhanced sanitation and social distancing at donation centers. Digital tools, such as mobile applications and online platforms, emerged as essential innovations to engage donors, schedule appointments, and provide real-time updates on blood shortages. Moreover, the crisis highlighted the need for adaptive donor recruitment strategies and the development of national and regional blood reserve policies. The integration of these new tools and policies into routine practice has the potential to strengthen the resilience of blood donation systems against future disruptions.

Attracting and retaining young blood donors requires a multifaceted approach that resonates with their values and lifestyles. Education plays a pivotal role, with schools and colleges serving as ideal platforms to introduce the importance of blood donation through curriculum integration and on-campus drives. Messaging that emphasizes the tangible impact of donations—such as real-life stories of recipients—can create a strong emotional connection, motivating young people to take action.

Technology is another critical tool. Social media campaigns, gamified donor platforms, and apps for scheduling appointments and tracking donation impact make the process engaging and convenient. Positive donation experiences, including streamlined procedures, comfortable facilities, and personalized appreciation, further encourage repeat participation. Incentives such as discounts, tokens, or recognition through donor programs also help sustain interest.

Peer influence is particularly effective among younger audiences. Peer-led initiatives, group donation drives, and competitions foster a sense of community and normalize the act of giving. Dispelling myths about donation safety and highlighting health benefits, such as routine health checks during donation, can help alleviate hesitations.

**Figure 1:**
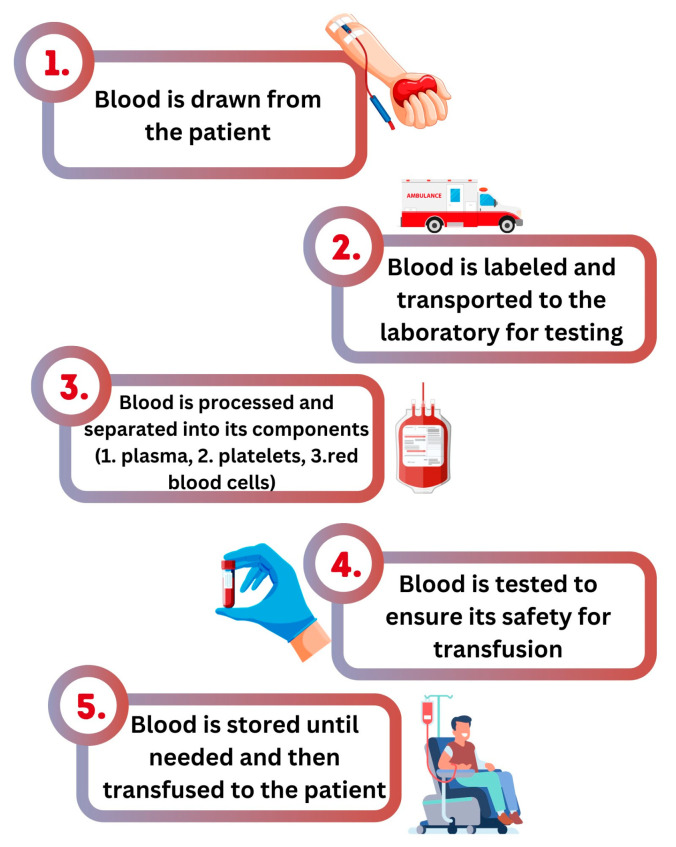
A schematic representation of the blood donation and transfusion process.

Long-term success lies in consistent engagement and messaging, using platforms like newsletters or social media to keep donation top-of-mind. Associating blood donation with annual events or community traditions can also anchor it as a valued social responsibility. By combining these strategies, organizations can cultivate a dedicated base of young donors, ensuring a sustainable blood supply for the future. The process of blood donation and transfusion is illustrated in Figure 1.

A reliable and sufficient blood supply serves as an important indicator of public health and access to healthcare services. Nearly 40 years after the World Health Assembly adopted Resolution WHA28.72/1975, which encouraged Member States to establish nationally coordinated blood services based on voluntary, unpaid donations, this issue remains a key focus in health service reform (Rome Declaration on Achieving Self-Sufficiency in Safe Blood and Blood Products, October 2013).

The WHO tailors its support to address the specific needs of individual countries, working in collaboration with the European Commission (EC), the Council of Europe/European Directorate for the Quality of Medicines and Health Care (CoE/EDQM), the European Centre for Disease Prevention and Control (ECDC), and other global partners. WHO advises that national policies for blood services should take a holistic approach, connecting health promotion, disease prevention, and medical care (in both primary care and hospitals) with community involvement.

Given the public’s close attention to the safety of blood supplies, there is a growing need to reshape national blood policies to focus on service integration, quality care, and patient safety as part of the broader goal of universal healthcare access. Additionally, blood components and plasma derivatives have been included in the WHO’s 18th Essential Medicines List.

## 3. Demographic Trends in Blood Donation

In conjunction with the work conducted by WHO, The European Blood Alliance (EBA) was established on 21 September 1998, by nine founding members: Belgium, England, Finland, France, Ireland, Luxembourg, the Netherlands, Portugal, and Scotland. As the primary representative of public and non-profit blood organizations in Europe, EBA offers trustworthy expertise and evidence-based information to both national and European institutions, as well as stakeholders in the broader global blood community. Currently, EBA has expanded its membership to 25, which includes Austria, Belgium, Croatia, Denmark, Estonia, Finland, France, Germany, Greece, Hungary, Iceland, Ireland, Italy, Latvia, Lithuania, Luxembourg, Malta, the Netherlands, North Macedonia, Norway, Portugal, Slovenia, Spain, Sweden, Switzerland, and the United Kingdom. The number of donated blood units in specific countries from 2010 to 2019 is summarized in Table 1.

The number of blood donations in EBA member countries over 10 years, with either an increasing or decreasing trend during the observed period is an epidemiologically significant indicator. A concerning downward trend is evident in most countries. A positive trend, i.e., an increase in the number of donated blood units, is present in only four countries: Croatia, Malta, France, and Latvia, while in other countries a decline in donated blood units ranging from −0.3% to −39.4% is observed. Five countries with the largest decrease in blood donations, with a decline greater than 30%, are Switzerland (−39.4%), Finland (−34.9%), the United Kingdom (−33.8%), Portugal (−33.2%), and Iceland (−32.7%). Given that these are economically developed countries with well-established healthcare systems and stable funding, it is necessary to systematically investigate the causes of this decline and develop sustainable public health programs to encourage blood donation motivation.

**Figure 2:**
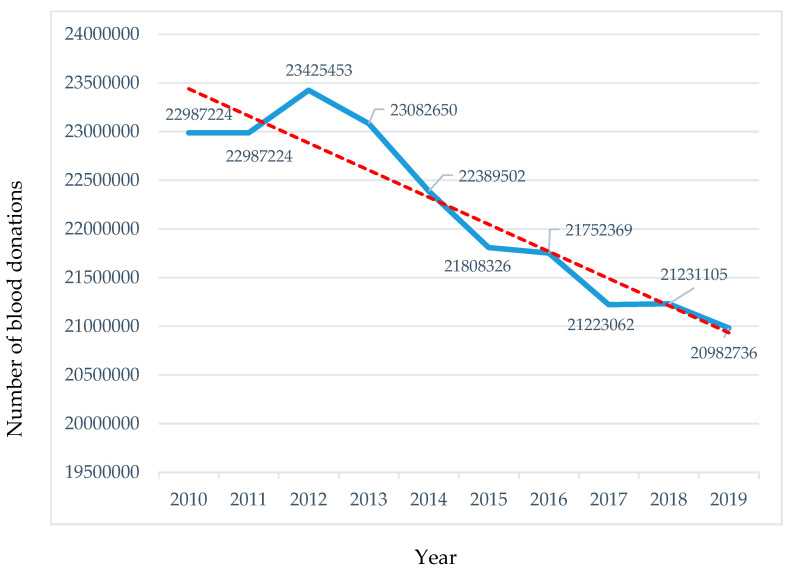
Total Number of Blood Donations (EBA Members), 2010–2019. Source: EBA Annual Report, 2020. (The red line represents the decreasing trend. The blue line represents the exact change).

Figure 2 illustrates the total number of blood donations in EBA member countries over time, highlighting a downward trend. The total number of donated units declined from 22,987,224 in 2010 to 20,982,736 in 2019, reflecting a decrease of 9.6%. Addressing this negative trend is critical, particularly as the demand for blood and blood products continues to grow due to advancements in diagnostic and therapeutic techniques across various medical disciplines [6].

### Blood Donation Statistics in the Republic of Croatia: A Decade in Review

Over the past decade, Croatia has experienced fluctuations in blood donation rates, reflecting broader trends in donor behavior and evolving healthcare demands. According to the Croatian Institute of Transfusion Medicine, Croatia has grown the number of registered blood donors very gradually. For example, in 2013, there were approximately 110,000 registered blood donors in Croatia, while this number decreased in 2023 to about 95,000. These may be influences caused by an aging population and a lifestyle change [9].

Still, the share of active blood donors in the overall population has remained steady at about 2–2.5%, which is very close to the average in most European Union countries. The Croatian Red Cross has been proactive in organizing regular blood drives and campaigns aimed at attracting as many new donors as possible, especially younger demographics [10]. Regardless of all these, Croatia still faces some difficulties in maintaining a secure blood supply, especially during peak demands and public health crises like COVID-19. The COVID-19 pandemic has drastically affected the rate of blood donation due to lockdowns and health concerns; the rate started recovering in recent years [11]. The epidemiological situation is similar in the European Union. Available data on the number of donors in EU Countries are summarized in Table 2.

Although more than half of the adult population meets the criteria for blood donation, it is estimated that only 5% of them donate blood, despite various campaigns encouraging participation [12]. Furthermore, only 1% of eligible adults donate blood regularly [13]. Regular blood donors are considered the safest donors, as their blood is repeatedly tested over time. To ensure a stable and safe blood supply, it is essential to develop sustainable blood donation promotion programs aimed at maintaining a steady donor base and recruiting new donors.

However, it is also important to acknowledge that the observed reduction in blood donations may correlate with a decline in demand. For example, there is ongoing debate among clinicians about transfusion practices, such as restrictive versus liberal blood transfusion strategies, which significantly influence the demand for blood. Studies indicate that restrictive transfusion practices, which limit transfusions to cases of severe anemia or critical clinical need, are increasingly favored and have led to reduced blood usage in many healthcare settings. Incorporating data on trends in blood demand and transfusion practices into blood donation strategies is crucial to align supply with current healthcare needs, making these programs not only cost-effective but also clinically relevant.

## 4. National Public Health Programs Promoting Blood Donation

Public health programs promoting blood donation offer numerous benefits to both the healthcare system and society. First, they ensure sufficient blood supplies by providing a stable and reliable source of blood and blood products essential for emergency interventions, surgeries, transfusions, chronic disease management, and other critical medical procedures. Second, these programs raise awareness and educate the public about the significance of blood donation, addressing misconceptions, demystifying the process, and highlighting how donations save lives. Through education, fear is reduced, and motivation to donate increases. Third, blood donation promotion programs contribute to improving public health by encouraging individuals to monitor their health regularly. Donors often undergo a “mini health check” before each donation, which includes a medical history review, a health questionnaire, and checks for blood pressure and hemoglobin levels. This process aids in the early detection of potential health issues, supporting the broader goal of enhancing population health.

However, while promoting blood donation can encourage individuals to engage in regular health checks, it is crucial to address potential risks associated with this practice. One significant concern is that some individuals may donate blood specifically to test for infectious diseases, such as HIV, at no cost. This creates a challenge, as infections in the early “window period” may be undetectable despite rigorous screening protocols, posing a risk to the blood supply. Blood donation centers prioritize donor safety and the integrity of the blood supply, emphasizing that blood donation should not be used as a substitute for proper medical testing. To mitigate this issue, public health messaging and education campaigns must clearly communicate that blood donation is not a diagnostic tool. Instead, individuals should be encouraged to seek confidential and comprehensive medical testing services if they suspect any health concerns. By addressing this risk, blood donation promotion programs can maintain public trust and ensure the safety of blood supplies.

Blood donation promotes the values of solidarity, community, and mutual support in society, which can increase overall social responsibility and a sense of collective well-being. This strengthens the social aspect of individual and population health. Moreover, supporting the healthcare system by ensuring stable blood supplies reduces the pressure on healthcare institutions, especially in crises such as natural disasters, pandemics, or increased demand for emergency medical interventions. Regular blood donation through well-organized programs contributes to the long-term sustainability of the healthcare system, reducing reliance on emergency blood appeals and improving the ability to plan medical procedures. In general, public health programs promoting blood donation foster greater trust in the healthcare system, protecting public health and saving lives [3,4,5,6].

### Characteristics of an Effective Public Health Program

A good public health program has several key characteristics that ensure its effectiveness and sustainability. A public health program must have clearly defined goals that are specific, measurable, achievable, relevant, and time-bound (SMART). For example, a blood donation promotion program might aim to increase the number of blood donors by 10% over the next 12 months in a specific area. An effective program should be supported by the government, healthcare institutions, and local communities. The support of relevant stakeholders facilitates program implementation and ensures the necessary resources. To achieve this, the program must involve the so-called triad of key stakeholders: decision-makers (often also the financiers), professionals, and the program’s users [7].

Every public health program should include educational campaigns that communicate the importance and benefits of the program. Education should be accessible to different age groups and social segments. In this, collaboration with various partners, such as the media (media campaigns), plays an important role, as it helps reach a wide target audience with minimal resources [8]. In addition to education, a successful program should motivate participation, whether through emotional, social, or economic incentives. For example, blood donors might be offered a free health check or a symbolic gift [9].

The program should be accessible to everyone, regardless of age, gender, socio-economic status, or place of residence, and it should include sensitive and marginalized groups. A good program must also have a system for monitoring progress and achieving goals. Evaluation allows for the adjustment of strategies based on results, thereby increasing the program’s effectiveness [10].

One of the challenges in creating public health programs is funding, as a sustainable financial model is needed. This can include public-private partnerships, government subsidies, or other financial mechanisms that ensure the program’s continuity. Moreover, since health priorities can change over time and across different areas, public health programs must be flexible enough to adapt to new challenges and changes in the population’s health status or societal needs. Of course, like all biomedical interventions, public health programs must adhere to ethical principles and ensure the protection of participants’ rights. Transparency and ethical conduct are essential for gaining public trust in the program [11].

While each country must tailor its public health programs to its specific context, with their effectiveness influenced by socio-cultural factors, it is essential to explore theoretically grounded principles to ensure the “anchoring” or sustainability of these programs. This includes proper design, implementation, and evaluation [12]. However, further research is needed to assess the effectiveness of public health programs and to identify the key goals that can enhance donor motivation [13].

## 5. Health Outcomes Associated with Blood Donation

Blood donation is an important public health practice not only for saving lives with the provision of blood products but also because it can have potential health benefits for donors themselves. Recent scientific knowledge on the health outcomes associated with blood donation, focusing on physical and psychological impacts, disease risk, and public health implications has been emphasized. These facts underscore the need for ongoing efforts to promote regular, voluntary blood donation and maintain a stable blood supply.

### 5.1. Physical Health

Different possible physical health benefits to donors through blood donation have been demonstrated previously. The most studied possible relation with the health of blood donors is the possible reduction in cardiovascular risk [14]. Regular blood donation can lower iron levels in the body, especially in men, hence reducing the risk of overload that was previously associated with cardiovascular diseases [15]. The excessive iron in the blood can thus lead to oxidative stress, which is considered to be of prime importance in atherosclerosis and other cardiovascular diseases. Blood donation is also associated with improved lipid profiles and a decrease in blood pressure, especially when iron levels in the blood are raised, as such donations contribute to maintaining cardiovascular health [14].

In addition, blood donation leads to a transient reduction of blood viscosity for up to several months, which in turn may lower the chances of clot formation [15]. This is crucial for individuals who are prone to venous thromboembolism or stroke [14]. The decline in blood viscosity has the effect of increasing blood flow, with the added potential of preventing heart attacks and other cardiovascular events [14,15,16]. In addition, regular blood donation has been shown to have potential health benefits in managing conditions like hereditary hemochromatosis and polycythemia vera [16]. This benefit is more plausible following multiple donations, since they cause prolonged reduced iron levels and hence better blood flow [14,15,16].

Blood donation can also reduce the risk of certain types of cancers, particularly in people with high levels of iron. High levels of iron have been associated with an increased risk of cancers like liver, colon, and lung cancer because excess iron is pro-oxidant in nature, leading to DNA damage and tumorigenesis. Some evidence suggests that frequent blood donation reduces iron storage and therefore lowers the oxidative burden on the body. While future research is required, there is indeed some possibility that frequent blood donation confers a protective effect, particularly against liver and colon cancers [17,18,19].

Besides these possible long-term benefits, blood donation provides regular health screening for donors. Every donation that one makes includes testing his or her hemoglobin levels, blood pressure, and general state of health, which may be an early detection for conditions such as hypertension or anemia. As part of health monitoring, donors receive information on their health that is not part of regular care [20].

Furthermore, the nature of these screenings could make regular donors more proactive about their health. This in turn could have wider implications for public health because conditions such as high blood pressure and iron imbalances can be treated early on, minimizing serious health problems later in life [21].

Therefore, blood donation, besides its critical role in saving lives through transfusion, provides a host of health benefits to the donors themselves and is hence one of the most important public health practices serving dual benefits to donors and recipients alike. While much has to be done along the lines of research to prove the full gamut of these benefits, the current evidence strongly supports the positive health outcomes of regular blood donation.

### 5.2. Psycological Health

Apart from physical health, blood donation shows different significant psychological and emotional benefits for donors. One of the most important mental health impacts reported among blood donors is the altruistic and social role one assumes and feels in helping others. This act of giving with no expectation of return conveys a sense of purpose linked to improved psychological well-being. Many donors have feelings of contentment and satisfaction, realizing that their blood can help save lives. Such good feelings are associated with reduced stress, heightened self-esteem, and better mental health overall [22,23].

Blood donation can be one avenue for an individual to contribute to society in a constructive manner. In fact, according to a review by Piliavin and Callero (2018), the psychological rewards of blood donation undoubtedly extend beyond the act of donating. The contribution of an improved sense of self-worth, which this temporary boost in self-esteem brings with it, together with the sense of belonging to a community of donors, is sometimes very important in the longer term. Many donors believe that they give not only to people in need but also to contribute to a higher and broader degree of humanitarian work. This perception helps people feel closer to society and strengthens the sense of community and social cohesion [24,25,26].

Blood donation also acts as one sort of psychological buffer against feelings of helplessness either in situations of crisis or natural disasters. Blood donation is an important opportunity when people are unable to participate directly in disaster management. In addition, blood donation itself serves as a positive way to express one’s empathy and the desire to help during a disaster. Many donors benefit psychologically from this act due to the assurance that they can help in such crucial moments. This improvement in mental comfort might reduce feelings of anxiety or any feeling of powerlessness while witnessing many crises [26].

Some studies also suggest that blood donation may serve as a means of reducing anxiety by taking one’s focus off of oneself and onto the welfare of others. This shifting of focus can disrupt the spirals of stress and promote positive emotional states. Lee et al., noted that donors exhibited lower levels of depression and anxiety symptoms than non-donors, perhaps through the positive psychological feedback loop fostered by helping others [26,27].

Apart from that, with the passage of time, regular blood donors will be engaged in routine and commitment to public health causes, which reinforces their psychological well-being. It can instill stability and a sense of purpose, something particularly needed when there are personal or professional stresses in one’s life. The emotional rewards from giving back through blood donation contribute to long-term mental resilience and overall life satisfaction [26,28].

Overall, blood donation is associated with a range of positive psychological and emotional benefits, from enhanced self-esteem and community connectedness to stress reduction and an increased sense of purpose. These benefits make voluntary blood donation at least as important a health service for the recipients as it is for the donors themselves in terms of emotional well-being and mental health. Blood donation is a life-saving act but it also depends on willingness to donate blood, depending on personal characteristics, beliefs, and motivations but also on the cultural context [29,30].

## 6. Challenges in Blood Donation Promotion Programs

Promoting blood donation faces several challenges. According to the WHO, 90% of eligible donors are not currently donating, highlighting a significant gap in participation.

Since blood donations have a short shelf life, a steady supply relies on regular donors. With blood shortages and aging populations, many regions are expanding the age limits for donors. Typically, the age range is 18 to 65, but in some European countries, it extends from 17 to 70. In certain places outside Europe, the limits range from 15 with parental consent to over 70 [31]. Regular blood donors, defined as those who donate at least twice yearly, are considered the safest donors. They undergo regular health checks and typically maintain a healthy lifestyle. The prevalence of blood-borne infections among donors varies from 0.001% to 7.5%, depending on the donor category. All donated blood is tested for hepatitis B, hepatitis C, syphilis, and HIV. This is why regular donors are considered the safest, as their blood is tested repeatedly, reducing the risk of error to a theoretical minimum. Blood donations found to carry those infections are discarded. One unit of donated whole blood can potentially save up to three lives, as its components can be separated and used for different patients [1,2,3,4].

Between 2008 and 2010, blood donations in 30 WHO European Region countries increased from 34.7 to 36.5 donations per 1,000 people. In Albania, voluntary, unpaid blood donations have increased fivefold since the launch of the South-eastern Europe Health Network’s blood safety project in 2004. Across Europe, blood donation rates vary widely, from 6 to 67.6 donations per 1,000 inhabitants, with Denmark having the highest rate. To be self-sufficient in blood supplies, a country typically needs an average of 20–25 regular donors per 1,000 people, though needs vary based on local disease patterns and available technologies [8].

The global shortage of blood donors can have serious consequences for treatment and diagnostics, affecting many medical interventions [32]. The main causes of this shortage lie in the lack of awareness among people about the need for blood donation, a lack of understanding of the process, and the lack of sustainable strategies to encourage donation. To achieve its goals and purposes, public health has at its disposal various public health tools that are used following scientific public health methodology.

One of these tools, which is the simplest to implement, is following the health calendar of WHO and marking days significant for community health, such as World Health Day, World Heart Day, and others. The goal of these observances is to draw the attention of the public, media, and authorities to a specific public health priority, offer improvement solutions, raise awareness about a particular issue, and encourage its resolution. Additionally, these efforts aim to increase the level of responsibility of individuals and the population for their health.

Advances in medicine increase the demand for blood and blood products. High blood consumption today could result in shortages in the future, as many medical interventions, such as surgeries, injury treatments, and cancer therapies, often require large quantities of blood. Therefore, everyone must recognize the importance of donating blood [33].

Education and promotion of blood donation can be carried out at various levels and through different activities involving multiple key stakeholders. To create a sustainable blood donation promotion program, it is necessary to systematically investigate the motives and reasons for donating (or not donating) blood in each population, as these motives may vary due to cultural differences [30]. Public awareness and education include campaigns and programs that promote blood donation and educate the public about the importance of blood donation, the donation process, and the safety measures in place. They also raise awareness about the ongoing need for blood and the benefits of regular donations. Community Involvement including blood donation fosters a sense of community and altruism. It encourages people to contribute to the well-being of their fellow citizens. Community-based blood drives and donor recognition programs help build a culture of regular blood donation.

Voluntary blood donation is the most cost-effective way to ensure a steady supply of safe blood. It reduces the need for expensive recruitment and compensation schemes associated with paid donation systems. It also minimizes the costs associated with treating transfusion-transmitted infections. Creating sustainable programs for promoting blood donation ensures a continuous and reliable donor base. These programs can include educational initiatives in schools and workplaces, partnerships with community organizations, and the use of social media to reach a broader audience.

## 7. Regulatory Framework and Safety Standards: Technological Innovations in Blood Collection and Storage

Recent advancements in blood collection and storage technologies have greatly enhanced safety, productivity, and blood storage. The newest methodologies for testing infectious diseases and techniques for preserving the blood components are major ways to ensure security in the supply of blood. Notable among recent technological changes to blood collection have been improvements in blood processing with automated systems introduced with greater safeguards for efficiency and safety. Advanced centrifugation methods in blood processing techniques ensure more precise separations of plasma, platelets, and red blood cells. These methods optimize the yield and quality of each component to meet specific patient needs [34]. These systems further minimize the risk of contamination and human error, which is an important requirement for ensuring safety and integrity in the blood supply chain.

New methods of testing infectious diseases have also played a very key role in improving the safety of blood. Contemporary techniques of pathogen reduction technologies, together with nucleic acid testing, enable one to detect pathogens in donations of blood with more sensitivity and much earlier than before. While testing is one approach, for example, Pathogen Reduction Technologies (PRTs), which are advanced methods used to reduce the risk of transfusion-transmitted infections by inactivating a wide range of pathogens, including viruses, bacteria, parasites, and even some white blood cells that may cause adverse reactions, inactivate a broad range of harmful microorganisms in blood products, helping to sharply reduce the risk of transfusion-transmitted infections. In other words, testing and pathogen reduction methods have been correspondingly applied so that the blood supply could be kept safe [35]. Additionally, PRTs have significantly improved blood product storage and safety. These technologies were developed for a broad-spectrum pathogen inactivation action: viruses, bacteria, and parasites while having minimal or no effect on the respective blood component quality. PRTs have also been demonstrated to extend the shelf life of platelets and plasma, two components very susceptible to bacterial contamination due to their conditions of storage. Thus, PRTs form part of the management of the blood supply, especially in periods of increased demand or shortages, by allowing longer-term storage with maintained safety and efficacy. PRTs therefore represent a significant advancement in transfusion medicine, providing an added layer of protection against known and emerging infectious threats. While highly effective, PRTs do not entirely eliminate all pathogens and may have varying efficacy against different microorganisms. The process can also slightly reduce the functional quality of certain blood components, though these effects are generally considered acceptable in light of the safety benefits [36].

Along with PRTs, various innovations in blood storage have focused on prolonging its components’ shelf life and functionality as a vital constituent for transfusions. The main steps concerned the elaboration of advanced additive solutions that can preserve the viability of red blood cells and platelets over an extended period. These solutions have been formulated to maintain an optimum environment for the blood cells by maintaining their metabolic activity without degradation [37]. This means that the efficacy of a transfusion is going to be enhanced by reducing the chances of complications, hence increasing overall success rates related to blood transfusion. In this case, the additive solution helps stabilize pH and delivers nutrients that are necessary for the blood cells, consequently maintaining the integrity such that the blood cells remain functional throughout their shelf life [38].

Moreover, in terms of the development of materials used to manufacture blood storage bags, advanced plasticizers are used to minimize the leaching of harmful chemicals. Traditional plasticizers used in blood bags have been implicated in leaching chemicals into stored blood, which compromises its safety and quality. Newly developed plasticizers reduce the chances of leaching and increase the product’s compatibility with the blood components, allowing longer preservation of the quality of blood stored. This innovation extends the shelf life of blood products while enhancing the safety of transfusions, thereby protecting patients from exposition to harmful substances [39,40].

Long-term storage of the blood components without deterioration in quality is important in the event of disasters or multiple traumas, which require immediate access to adequate quantities of safe blood. These technological advances in the storage and preservation of blood allow blood banks and health professionals to manage their inventories more precisely, probably reducing the number of expired products that go to waste and ensuring timely access to lifesaving transfusions when required [41]. Improving inventory management by blood banks and health professionals would reduce the number of expired products that go to waste and ensure timely access to lifesaving transfusions when required [42,43]. The development of new storage options and pathogen reduction technologies continues to mark a new frontier in the world of transfusion medicine and translates into safer, more efficient blood transfusions worldwide.

## 8. Conclusions

Public health plays a key role in understanding the long-term impact of voluntary blood donation on health behaviors and responsible conduct. It also highlights the importance of blood donors as a specific social group vital to the healthcare system. To ensure a steady supply, it is essential to understand the socio-demographic structure of younger, new donors, as they contribute to the safety of blood products and healthcare system stability. Regular donors, whose blood is frequently tested, are the safest, minimizing the risk of infectious diseases.

Understanding the motivations and behaviors of blood donors helps shape public health strategies to increase and retain regular, healthy donors. This impacts the sustainability and quality of the healthcare system by reducing waiting lists for blood-dependent services. WHO emphasizes the importance of healthy lifestyles for disease prevention, and public health programs should promote healthy behaviors through education, especially targeting specific social groups. Sustainable blood donation programs, such as school and workplace initiatives, community partnerships, and social media campaigns, are crucial for maintaining a reliable donor base. These programs also improve donor health and support healthcare delivery. Despite the benefits, international blood donation rates are declining, especially in developed countries, due to aging populations and crises like COVID-19. Addressing these challenges requires sustainable public health programs focused on awareness, education, and social responsibility.

Technological advances in blood collection, storage, and safety have improved the blood supply, but continuous efforts are needed to recruit new donors, particularly from younger generations, and to sustain regular donor participation. Ultimately, voluntary blood donation is not just about saving lives but also about fostering community, solidarity, and social responsibility. Ongoing promotion through targeted campaigns, collaboration between healthcare systems, and modern technologies is vital for ensuring a safe and adequate blood supply for future generations. These efforts are fundamental to the long-term sustainability of healthcare systems, especially in emergencies, making voluntary blood donation a cornerstone of global public health.

## Figures and Tables

**Table 1:** Number of donated blood units by country over 10 years (from 2010 to 2019) and the decrease percentage during the observed period. (Source: EBA Annual Report 2020).

	2010	2011	2012	2013	2014	2015	2016	2017	2018	2019	% Change
Austria	431,770	419,575	392,195	374,526	362,809	359,954	352,024	359,055	356,871	350,397	−23.20%
Belgium	622,197	629,238	627,830	578,701	546,478	551,710	558,698	551,058	563,336	593,316	−4.90%
Croatia	177,355	180,266	182,068	183,688	183,716	195,599	197,294	104,474	100,949	193,650	8.40%
Denmark	339,094	313,401	296,833	290,400	290,400	275,051	278,614	278,639	278,776	281,500	−20.50%
Estonia	58,729	59,676	60,057	61,234	60,506	59,013	57,417	55,057	53,486	56,787	−3.40%
Finland	270,913	264,607	246,808	227,604	220,980	211,536	205,068	204,946	206,609	200,822	−34.90%
France	2,875,921	3,007,412	3,104,295	2,833,351	2,796,308	2,892,286	2,938,409	2,890,146	2,997,293	2,966,603	3.10%
Germany	7,495,452	7,575,313	7,447,253	7,365,562	7,206,860	6,875,363	6,760,345	6,741,597	6,477,225	6,565,859	−14.20%
Greece		570,780	569,491	584,088	558,349	549,755	555,206	571,791	569,207	569,207	−0.30%
Hungary	418,794	429,171	454,978	415,339	409,391	403,916	397,262	397,262	385,555	395,659	−5.80%
Iceland	15,340	13,780	13,401	13,663	12,318	11,608	11,393	11,531	11,221	11,556	−32.70%
Ireland	159,390	154,024	149,614	143,778	145,325	144,083	136,607	136,607	140,420	141,791	−12.40%
Italy	3,199,787	3,221,131	3,221,131	3,198,806	3,081,777	3,061,479	3,036,634	3,048,216	3,048,216	3,003,072	−6.60%
Latvia	57,045	57,752	55,253	53,942	56,528	59,029	57,448	57,448	56,775	59,784	4.60%
Lithuania	77,256	87,971	88,749	61,254	66,147	70,774	73,710	73,652	69,177	72,865	−6.00%
Luxembourg	24,055	24,055	20,631	24,191	23,904	23,657	23,657	24,172	22,486	21,555	−11.60%
Malta	15,096	16,960	17,424	17,024	17,457	17,427	18,152	16,363	16,547	17,528	13.90%
Netherlands	883,346	885,971	819,301	755,833	721,012	720,251	726,273	720,464	724,002	727,462	−21.40%
Norway	217,907	217,907	217,907	222,954	223,455	204,065	200,888	193,723	190,408	188,814	−15.40%
Portugal	419,574	416,749	392,136	362,372	353,829	337,899	334,022	324,053	318,951	315,045	−33.20%
Slovenia	98,302	98,302	95,442	93,790	89,226	88,751	91,280	90,600	88,100	86,230	−14.00%
Spain	1,823,315	1,823,315	1,757,940	1,698,097	1,676,308	1,706,973	1,698,759	1,686,463	1,660,143	1,660,143	−9.80%
Sweden	559,999	546,770	516,577	504,920	487,848	480,487	462,261	446,979	436,767	435,846	−28.50%
Switzerland	368,167	371,016	360,011	344,174	330,495	306,235	294,969	282,546	270,827	264,179	−39.40%
United Kingdom	2,376,410	2,038,300	1,973,313	1,978,198	1,898,882	2,080,013	1,690,200	1,801,790	1,775,459	1,775,459	−33.80%

**Table 2:** Number of Blood Donors in European Union Countries, 2014 [8].

Member State	Population	Number of Blood Donors	Ratio of Blood Donors in the General Population
Austria	8,951,520	n/a	n/a
Belgium	11,521,238	289,918	2.52%
Bulgaria	6,838,937	159,782	2.34%
Croatia	3,888,529	94,168	2.42%
Czechia	10,712,000	250,000	2.33%
Denmark	5,873,420	183,409	3.12%
Estonia	1,331,796	28,211	2.12%
Finland	5,550,000	113,006	2.04%
France	66,410,000	1,578,827	2.38%
Germany	83,408,554	n/a	n/a
Greece	10,370,747	n/a	n/a
Hungary	9,800,000	n/a	n/a
Ireland	4,986,526	72,791	1.46%
Italy	59,236,213	1,653,268	2.79%
Latvia	1,866,942	33,323	1.78%
Lithuania	2,800,000	54,120	1.93%
Luxembourg	634,730	12,698	2.00%
Malta	442,784	n/a	n/a
Netherlands	17,475,415	384,352	2.20%
Poland	38,538,447	615,927	1.60%
Portugal	10,344,802	235,178	2.27%
Cyprus	1,215,584	78,475	6.46%
Romania	19,127,774	313,843	1.64%
Slovakia	5,447,247	119,269	2.19%
Slovenia	2,101,000	62,000	2.95%
Spain	47,160,415	1,133,131	2.40%
Sweden	9,800,000	198,709	2.03%

## Data Availability

No new data were created or analyzed in this study. Data sharing is not applicable to this article.

## References

[1] Miller A. (2023). Blood Donation and Public Health—The Broader Impact on Communities. Donate Blood–The Blood Connection.

[2] Guidelines Review Committee (2010). Towards 100% Voluntary Blood Donation: A Global Framework for Action.

[3] Dorle A., Gajbe U., Singh B.R., Noman O., Dawande P. (2023). A review of awareness about blood donation through various effective and practical strategies. Cureus.

[4] Zeger G., Selogie E., Shulman I.A. (2007). Blood donation and collection. Blood Banking and Transfusion Medicine.

[5] Dejene M., Tefera A., Dires A., Gedamu S., Getachew Y., Ademe S. (2021). Low Blood Donation Practice of Health Sciences College Students in Northeast Ethiopia: A Cross-Sectional Study. J. Blood Med..

[6] WHO Blood Safety and Availability. https://www.who.int/news-room/fact-sheets/detail/blood-safety-and-availability.

[7] Wittock N., Hustinx L., Bracke P., Buffel V. (2017). Who donates? Cross-country and periodical variation in blood donor demographics in Europe between 1994 and 2014. Transfusion.

[8] Piersma T.W., Merz E.M. (2019). (Non-)donor demographics, donation willingness, and the donor career. Transfusion.

[9] HZTM HZTM. https://hztm.hr/.

[10] Hrvatski Crveni Križ. https://www.hck.hr/.

[11] European Blood Alliance European Blood Alliance. https://europeanbloodalliance.eu/.

[12] Hogan B., Hershey L., Hogan R. (2008). Using sponsorship to improve the success of blood drive donations. Transfusion.

[13] France J.L., France C.R., Himawan L.K. (2007). A path analysis of intention to resonate among experienced blood donors: An extension of the theory of planned behavior. Transfusion.

[14] Quee F.A., Peffer K., Ter Braake A.D., Van Den Hurk K. (2022). Cardiovascular benefits for blood donors? A systematic review. Transfus. Med. Rev..

[15] Bani-Ahmad M.A., Khabour O.F., Gharibeh M.Y., Alshlool K.N. (2017). The impact of multiple blood donations on the risk of cardiovascular diseases: Insight of lipid profile. Transfus. Clin. Biol..

[16] Kebalo A.H., Gizaw S.T., Gnanasekaran N., Areda B.G. (2022). Lipid and Haematologic Profiling of Regular Blood Donors Revealed Health Benefits. J. Blood Med..

[17] Thorpe R., Masser B., Coundouris S.P., Hyde M.K., Kruse S.P., Davison T.E. (2024). The health impacts of blood donation: A systematic review of donor and non-donor perceptions. Blood Transfus..

[18] Vasanthi H.R., Mukherjee S., Das D.K. (2021). Retraction Notice To: Potential health benefits of broccoli: A chemico-biological overview. Mini Rev. Med. Chem..

[19] Fonseca-Nunes A., Jakszyn P., Agudo A. (2014). Iron and cancer risk—A systematic review and meta-analysis of the epidemiological evidence. Cancer Epidemiol. Biomark. Prev..

[20] Myers D.J., Collins R.A. (2024). Blood Donation. StatPearls.

[21] Piliavin J.A. (1991). Giving Blood: The Development of an Altruistic Identity.

[22] Hoogerwerf M.D., Veldhuizen I.J., de Kort W.L., Frings-Dresen M.H., Sluiter J.K. (2015). Factors associated with psychological and physiological stress reactions to blood donation: A systematic review of the literature. Blood Transfus..

[23] Rigas A.S., Skytthe A., Erikstrup C., Rostgaard K., Petersen M.S., Hjalgrim H., Ullum H., Kyvik K.O., Pedersen O.B. (2019). The healthy donor effect impacts self-reported physical and mental health—Results from the Danish Blood Donor Study (DBDS). Transfus. Med..

[24] Williams L.A., Masser B., Van Dongen A., Thijsen A., Davison T. (2017). The emotional psychology of blood donors: A time-course approach. ISBT Sci. Ser..

[25] Piliavin J.A., Callero P.L., Evans D.E. (1982). Addiction to altruism? Opponent-process theory and habitual blood donation. J. Pers. Soc. Psychol..

[26] Balaskas S., Koutroumani M., Rigou M. (2024). The mediating role of emotional arousal and donation anxiety on blood donation intentions: Expanding on the theory of planned behavior. Behav. Sci..

[27] Lee Y., Park H., Jee H.J. (2020). Psychological characteristics and associations between living kidney transplantation recipients and biologically related or unrelated donors. BMC Nephrol..

[28] Fishman L. The Surprising Benefits of Donating Blood. NewYork-Presbyterian.

[29] Huis in‘t Veld E.M.J., de Kort W.L.A.M., Merz E.M. (2019). Determinants of blood donation willingness in the European Union: A cross-country perspective on perceived transfusion safety, concerns, and incentives. Health Soc. Care Community.

[30] Gillespie T.W., Hillyer C.D. (2002). Blood donors and factors impacting the blood donation decision. Transfus. Med. Rev..

[31] Mohammed S., Essel H.B. (2018). Motivational factors for blood donation, potential barriers, and knowledge about blood donation in first-time and repeat blood donors. BMC Hematol..

[32] Torrent-Sellens J., Salazar-Concha C., Ficapal-Cusí P., Saigí-Rubió F. (2021). Using digital platforms to promote blood donation: Motivational and preliminary evidence from Latin America and Spain. Int. J. Environ. Res. Public Health.

[33] Getie A., Wondmieneh A., Bimerew M., Gedefaw G., Demis A. (2020). Blood donation practice and associated factors in Ethiopia: A systematic review and meta-analysis. BioMed Res. Int..

[34] Aliaga A.I.P., Labata B.G., Manlangit L.C., Manalastas M.A.C., Natividad A.J.R. (2020). Motivational and barrier factors of voluntary blood donation: A cross-sectional study among university students in the Philippines. Philipp. J. Health Res..

[35] Liu H., Wang X. (2021). Pathogen reduction technology for blood component: A promising solution for prevention of emerging infectious disease and bacterial contamination in blood transfusion services. J. Photochem. Photobiol..

[36] Rebulla P., Prati D. (2022). Pathogen reduction for platelets—A review of recent implementation strategies. Pathogens.

[37] Rajashekaraiah V., Rajanand M.C. (2022). Platelet storage: Progress so far. J. Thromb. Thrombolysis.

[38] Tran L.N.T., González-Fernández C., Gomez-Pastora J. (2024). Impact of different red blood cell storage solutions and conditions on cell function and viability: A systematic review. Biomolecules.

[39] Macopharma Non-Phthalate Plasticizer DEHT. https://www.macopharma.com/wp-content/uploads/2020/10/Non-phthalate-plasticizer-DEHT.pdf.

[40] Morishita Y., Nomura Y., Fukui C., Kawakami T., Ikeda T., Mukai T., Yuba T., Inamura K., Yamaoka H., Miyazaki K. (2017). Pilot study on novel blood containers with alternative plasticizers for red cell concentrate storage. PLoS ONE.

[41] Rabcuka J., Blonski S., Meli A., Sowemimo-Coker S., Zaremba D., Stephenson D., Dzieciatkowska M., Nerguizian D., Cardigan R., Korczyk P.M. (2022). Metabolic reprogramming under hypoxic storage preserves faster oxygen unloading from stored red blood cells. Blood Adv..

[42] Ontario Regional Blood Coordinating Network Inventory Management Toolkit. https://transfusionontario.org/wp-content/uploads/2020/08/InventoryManagementToolkit_rev2017-3.pdf.

[43] Godin G., Conner M., Sheeran P., Bélanger-Gravel A., Germain M. (2007). Determinants of repeated blood donation among new and experienced blood donors. Transfusion.

